# Supplementary material for: Melt volume at Atlantic volcanic rifted margins controlled by depth-dependent extension and mantle temperature
Source: Nat Commun. 2021 Jun 23;12:3894. doi: 10.1038/s41467-021-23981-5 (PMC8222230; doi:10.1038/s41467-021-23981-5)
Supplement: Supplementary file 1 — Supplementary Information [file 41467_2021_23981_MOESM1_ESM.docx]

*Supplementary Information*

Melt volume at Atlantic volcanic rifted margins controlled by depth-dependent extension and mantle temperature

Gang Lu^1,*^ and Ritske S. Huismans^1^

^1^*Department of Earth Science, Bergen University, Bergen, N-5007, Norway*

**Corresponding author:* gang.lu@geo.uib.no

Contents:

Supplementary Figures 1-7

Supplementary Tables 1-3

Supplementary References

**
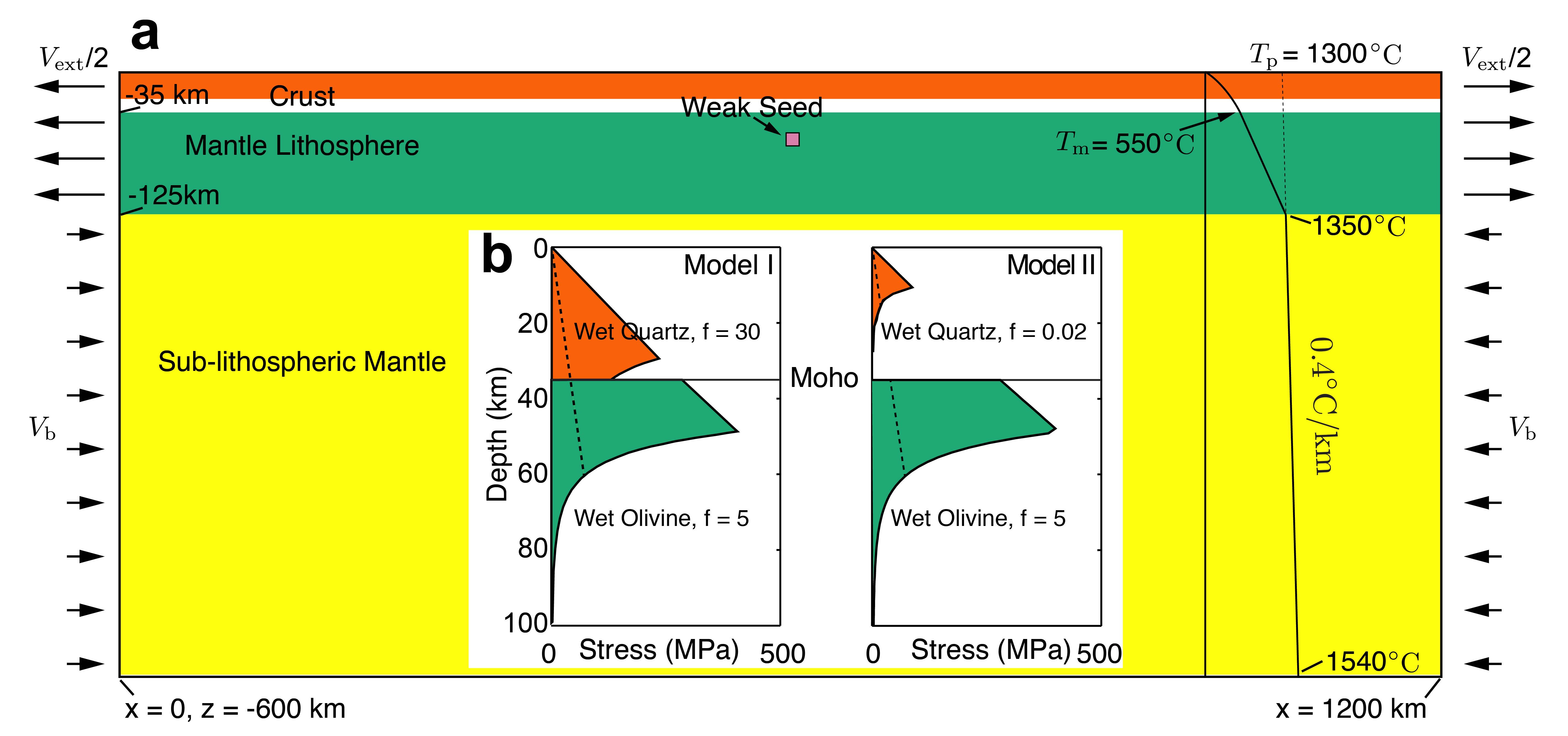
**

**Supplementary Figure 1.** Model configuration. **a**, Composition and temperature setup for the reference model. Model dimension is 1200 km wide by 600 km high, with 25-km upper crust (orange), 10-km lower crust (white), 90-km mantle lithosphere (green), and 475-km sub-lithospheric mantle (yellow). The upper crust and lower crust have the same properties. An initial weak seed (pink) is imposed in the centre to localize deformation. The lithosphere is extended at a full velocity of *V*_ext_ = 1.5 cm/yr. The laterally homogeneous initial temperature (black line) is constrained by potential temperature (*T*_p_ = 1300 °C), Moho temperature (*T*_m_ = 550 °C), and a constant adiabatic gradient of 0.4 °C/km in the sub-lithospheric mantle. In the crust, the initial temperature is analytically configured by $T=-\frac{A_{r}}{2k}\left( z-z_{m} \right)z+\frac{T_{m}}{z_{m}}z$, with crustal heat production $A_{r}=2\frac{k}{z_{m}}(\frac{T_{m}}{z_{m}}-\frac{T_{l}-T_{m}}{z_{l}-z_{m}})$ such that the lithosphere is initially in steady state, where *k* is thermal conductivity and *z*_m_, *z*_l_ are depths of Moho and base lithosphere. See Supplementary Table 1 for parameters. **b**, Strength profiles for Model I (strong crust) and Model II (weak crust). Dashed lines show strength profiles after strain weakening.

**
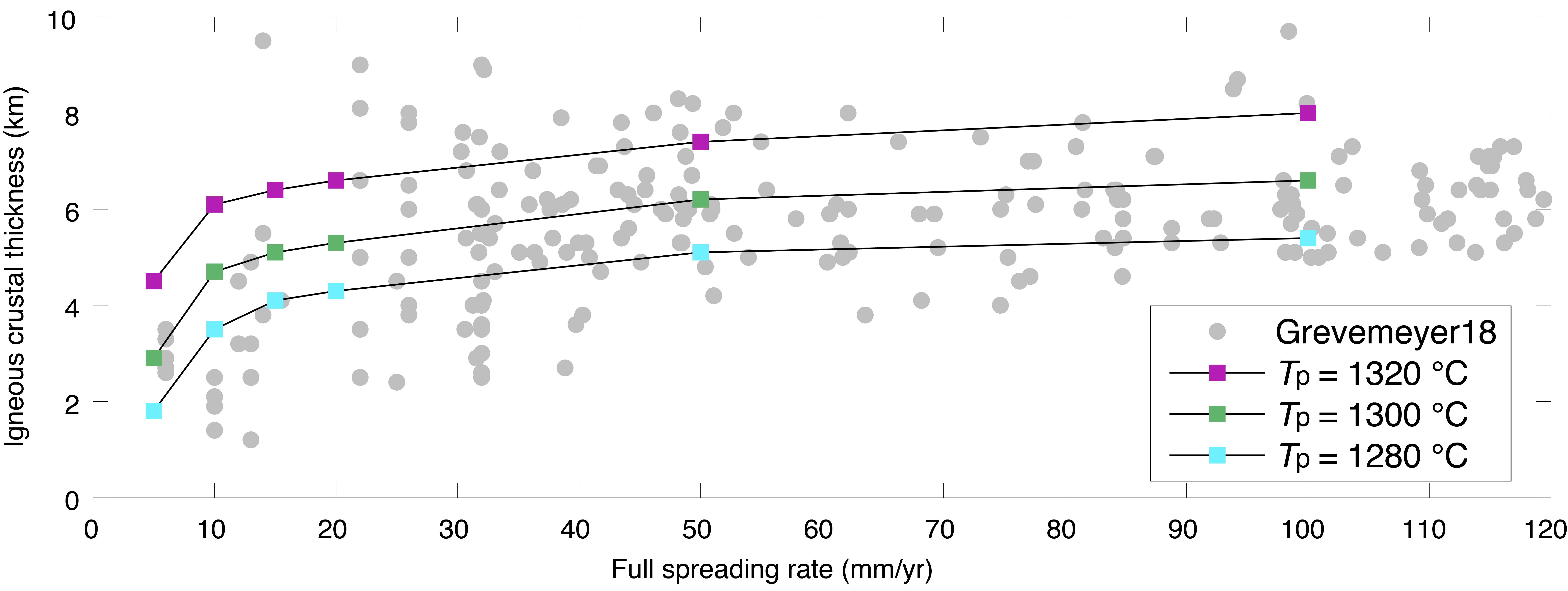
**

**Supplementary Figure 2. Benchmark of melt generation model**. Grey dots show observed oceanic crust thickness away from hotspots^1^. Colour squares indicate thickness of predicted oceanic crust at various spreading rates and mantle temperatures. Predicted oceanic crustal thickness is measured at mature spreading when igneous crust reaches steady state thickness.

**
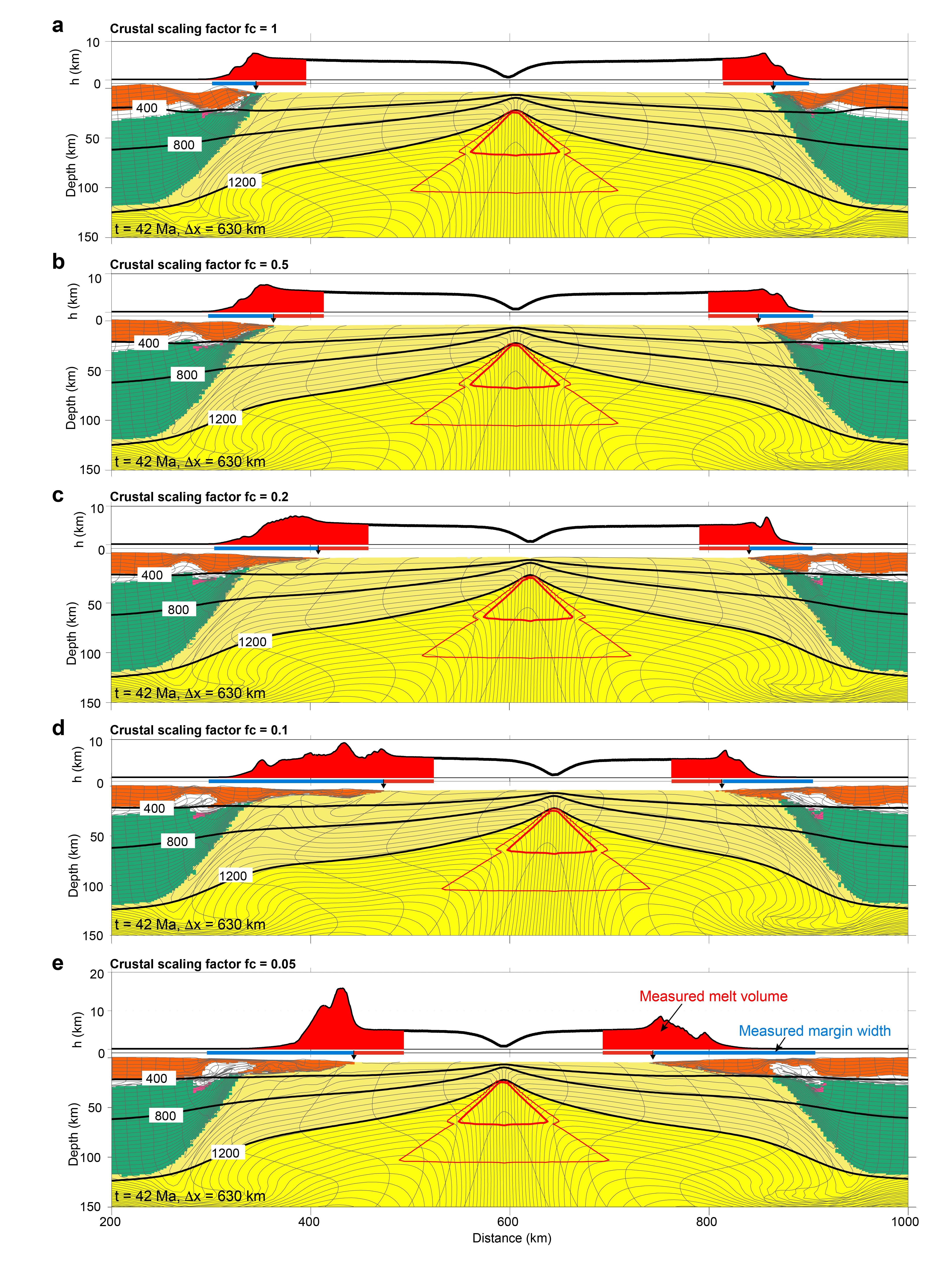
**

**Supplementary Figure 3. Melt production measurement for models with varying crustal strength. a-e,** Snapshots of models with decreasing crustal scaling factor (*f*_c_) from 1 to 0.05, leading to increasing margin width and melt thickness. All models shown are at the same amount of extension of Δx = 630 km. Red filled areas show domains for total melt volume (*V*^*^) calculation. Black arrows indicate COB. Blue bars indicate margin width measurement. Red bars indicate 50-km-wide initial oceanic spreading section that is included in melt volume measurement.

**
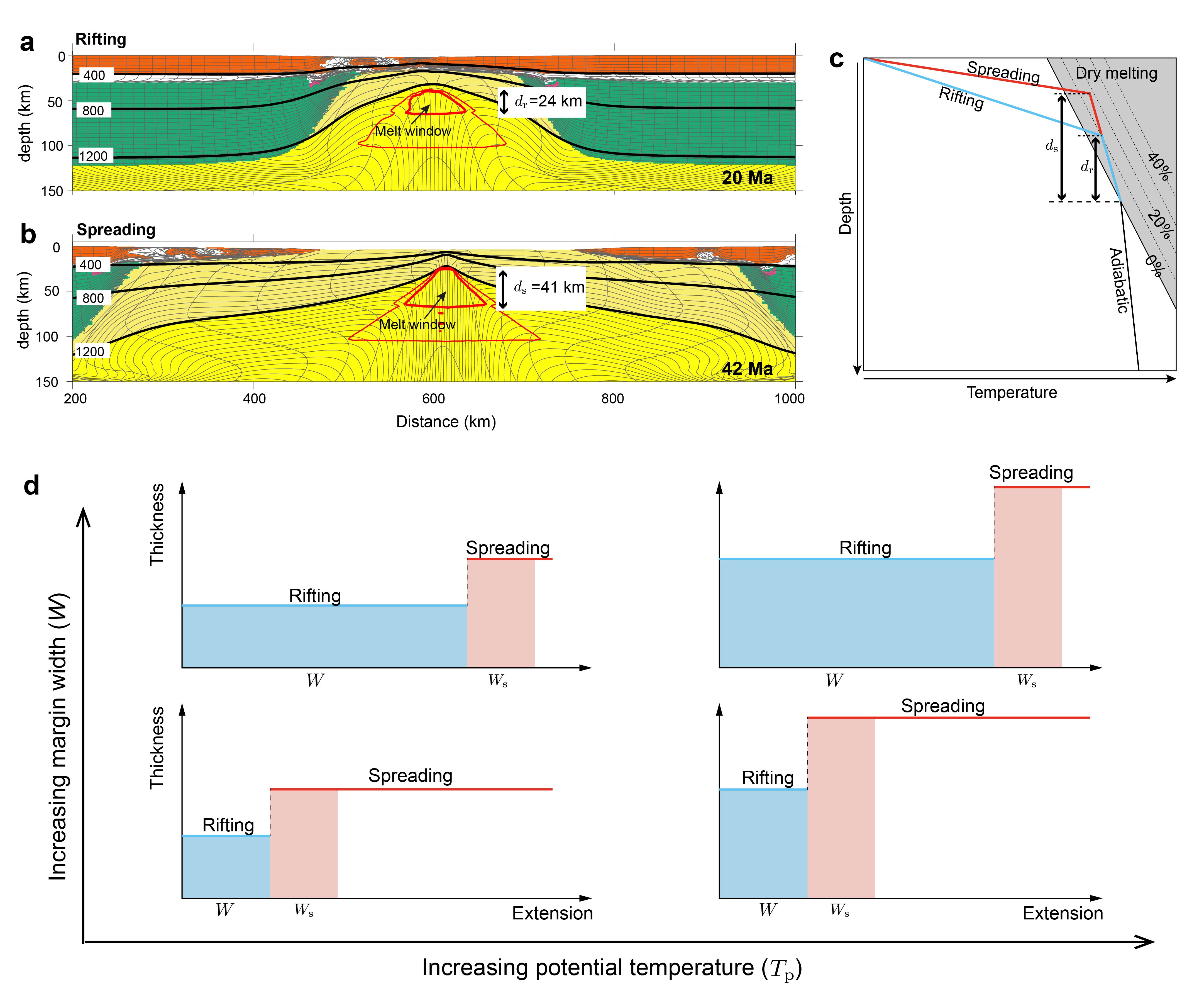
**

**Supplementary Figure 4. Semi-analytical scaling law analysis.** **a**, **b**, Snapshots showing melt windows during rifting (**a**) and spreading (**b**) for Model II. Red lines show contours of incremental melt fraction, with the thick lines indicating dry melting domain. *d*_r_ and *d*_s_ indicate heights of melt window with dry melting during continental rifting and during oceanic spreading. **c**, Melt parameterization model with conceptual geotherms for rifting (blue) and spreading (red). The extent of melting is proportional to the heights of melt windows during rifting (*d*_r_) and spreading (*d*_s_). **d**, Conceptual explanation for the semi-analytical prediction. Blue and red lines indicate melt volume per unit distance of extension (i.e. igneous crustal thickness) during rifting and spreading, respectively. *W* is margin width. *W*_s_ = 100 km is the total extension of the initial spreading section. Shadowed region show total melt volume measured in this study. Increased mantle temperature results in larger melt thickness, while longer rifting phase (blue line) leads to larger margin width. Both may increase melt volume during rifting (blue region).

**
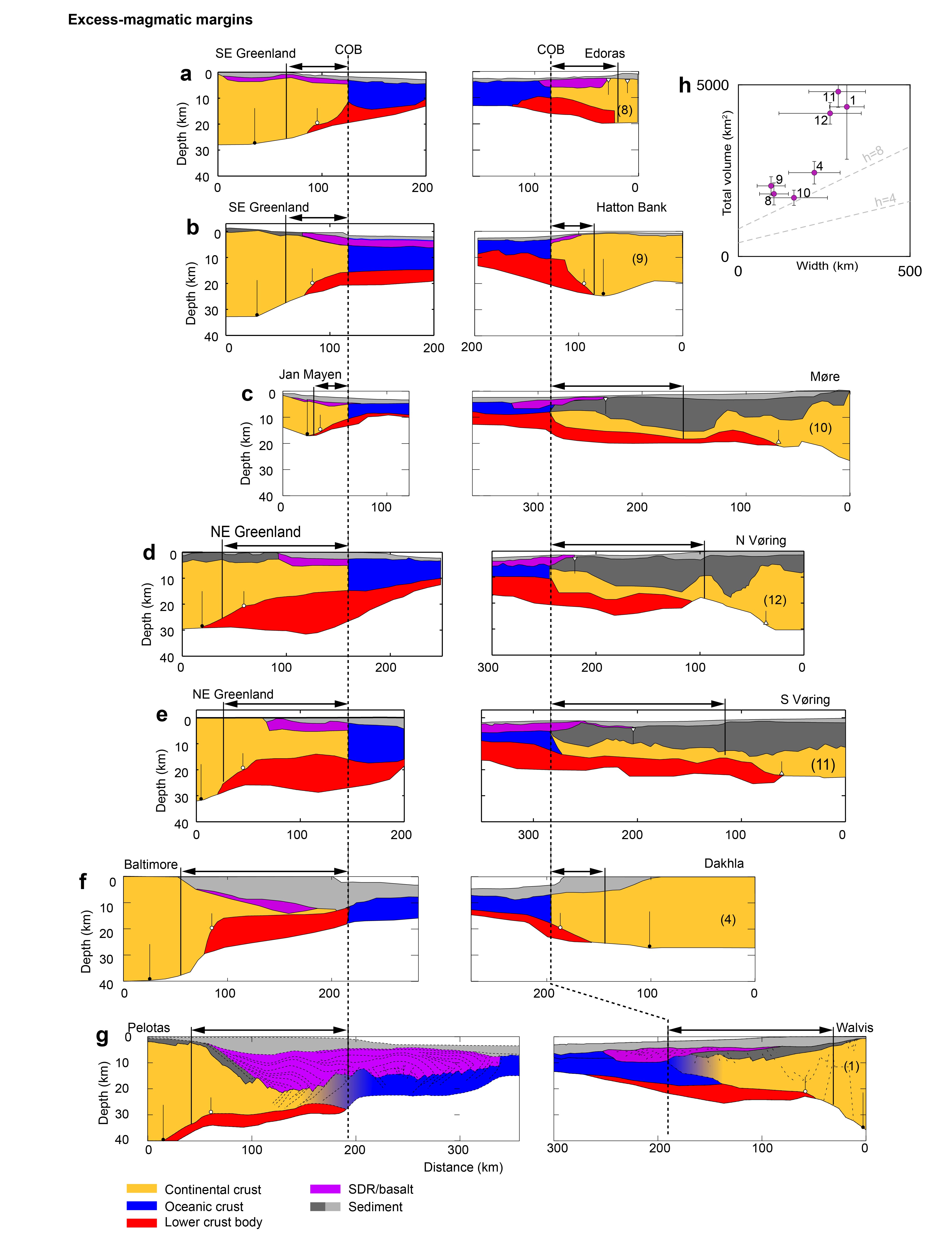
**

**Supplementary Figure 5. Melt volume measurement of excess-magmatic margins.** **a-g**, Conjugate crustal cross sections for excess magmatic margins ordered increasing in width from top to bottom. Oceanic crust (blue), underplated magmatic bodies (red) and extrusive magmatism (purple) are differentiated following ref. ^2^. (**a**) SE Greenland – Edoras^3–7^, (**b**) SE Greenland – Hatton Bank^3,5,6,8–11^, (**c**) Jan Mayen – Møre^2,5,6,12–14^, (**d**) NE Greenland – N Vøring^2,5,6,13,15–17^, (**e**) NE Greenland – S Vøring^2,5,6,13,15,16,18–20^, (**f**) Baltimore – Dakhla^21–25^, and (**g**) Pelotas – Walvis^26–33^ conjugate margins. Dashed lines indicate COB. Numbers mark the id of conjugate margins (see Fig. 6 for locations). Arrows indicate margin width. For the NE Greenland margins, we used the interpretation by ref. ^5^, consistent with paleo-geographic reconstructions for the N Atlantic, although there are alternative views to location of the COB^2,5,6,15,16,34^. For the Jan Mayen (10) and NE Greenland (14) margin sections where the maximum Moho depth is less than 20 km, we define the crustal taper between the location of maximum depth and the location where Moho reaches 15 km depth. For the Edoras (8) section crustal taper is not well constrained because the Moho is flat and there is no data on crustal thickness further landward^4^. For this margin we have therefore used the occurrence of extrusive magmatism and the location of maximum topography within the section, which likely gives a lower limit of margin width. **h**, Estimated melt volume and width with uncertainty for each conjugate margin pair. Dashed lines show semi-analytical prediction for oceanic crustal thickness *h*_oc_ = 4 km and *h*_oc_ = 8 km delimiting ‘normal’ magmatic productivity. Note that all conjugate margin pairs shown here show excess magmatic productivity with respect to this reference.

**
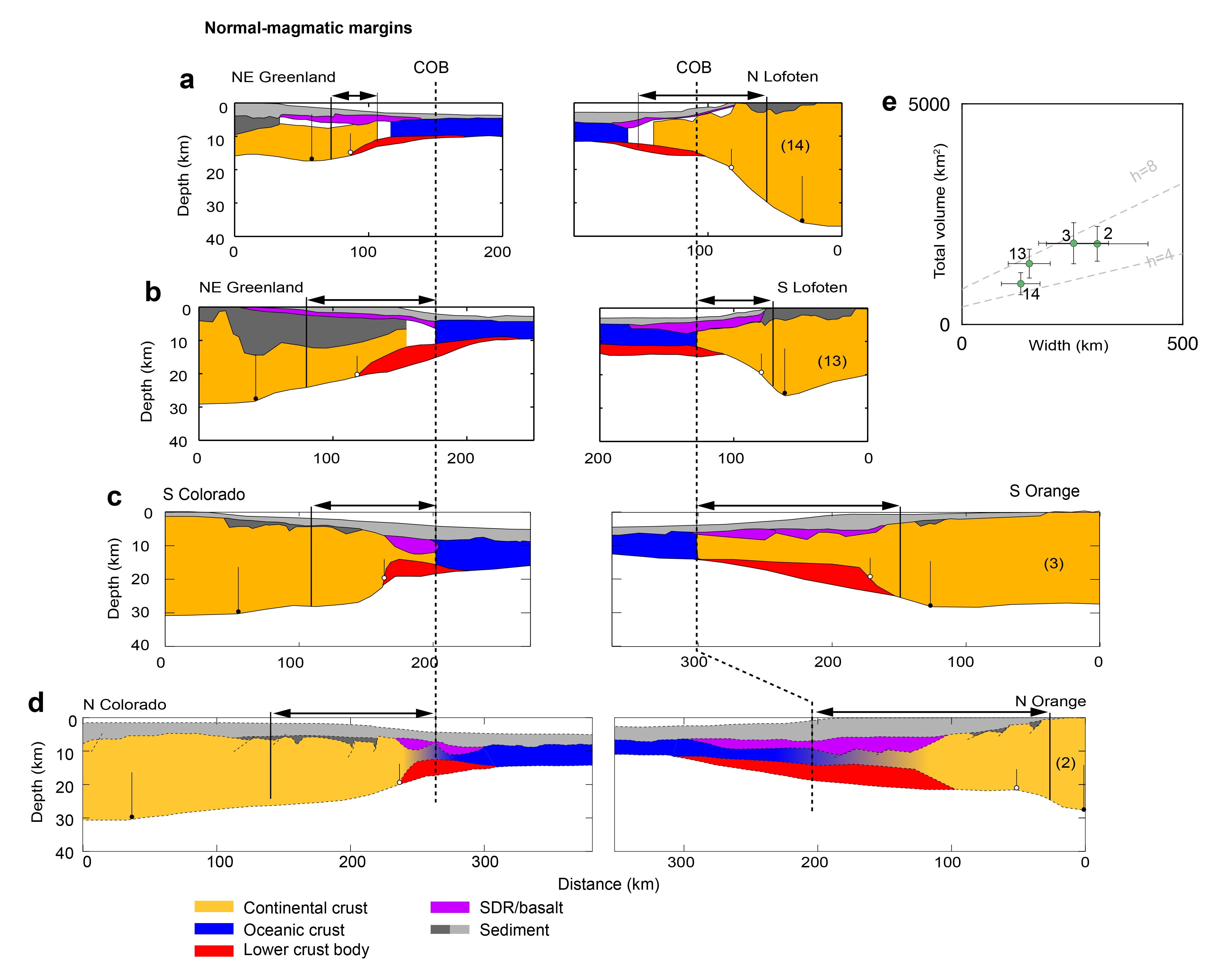
**

**Supplementary Figure 6. Melt volume measurement of normal-magmatic margins.** **a-d**, Conjugate crustal cross sections for normal magmatic margins ordered increasing in width from top to bottom. Oceanic crust (blue), underplated magmatic bodies (red) and extrusive magmatism (purple) are differentiated following ref. ^2^. (**a**) NE Greenland – N. Lofoten^2,6,13,16,35^, (**b**) NE Greenland – S. Lofoten^2,5,6,13,16,36^, (**c**) S. Colorado –S. Orange^26,37–43^, (**d**) N. Colorado – N. Orange^26,33,37,41^ conjugate margins. Numbers mark the id of conjugate margins (see Fig. 6 for locations). Arrows indicate margin width. **e**, Estimated melt volume and width for each conjugate margin pair. Dashed line shows semi-analytical prediction with *h*_oc_ = 4 km and *h*_oc_ = 8 km. Note that all conjugate margin pairs shown here are within the range of normal magmatic productivity for oceanic spreading systems.

**
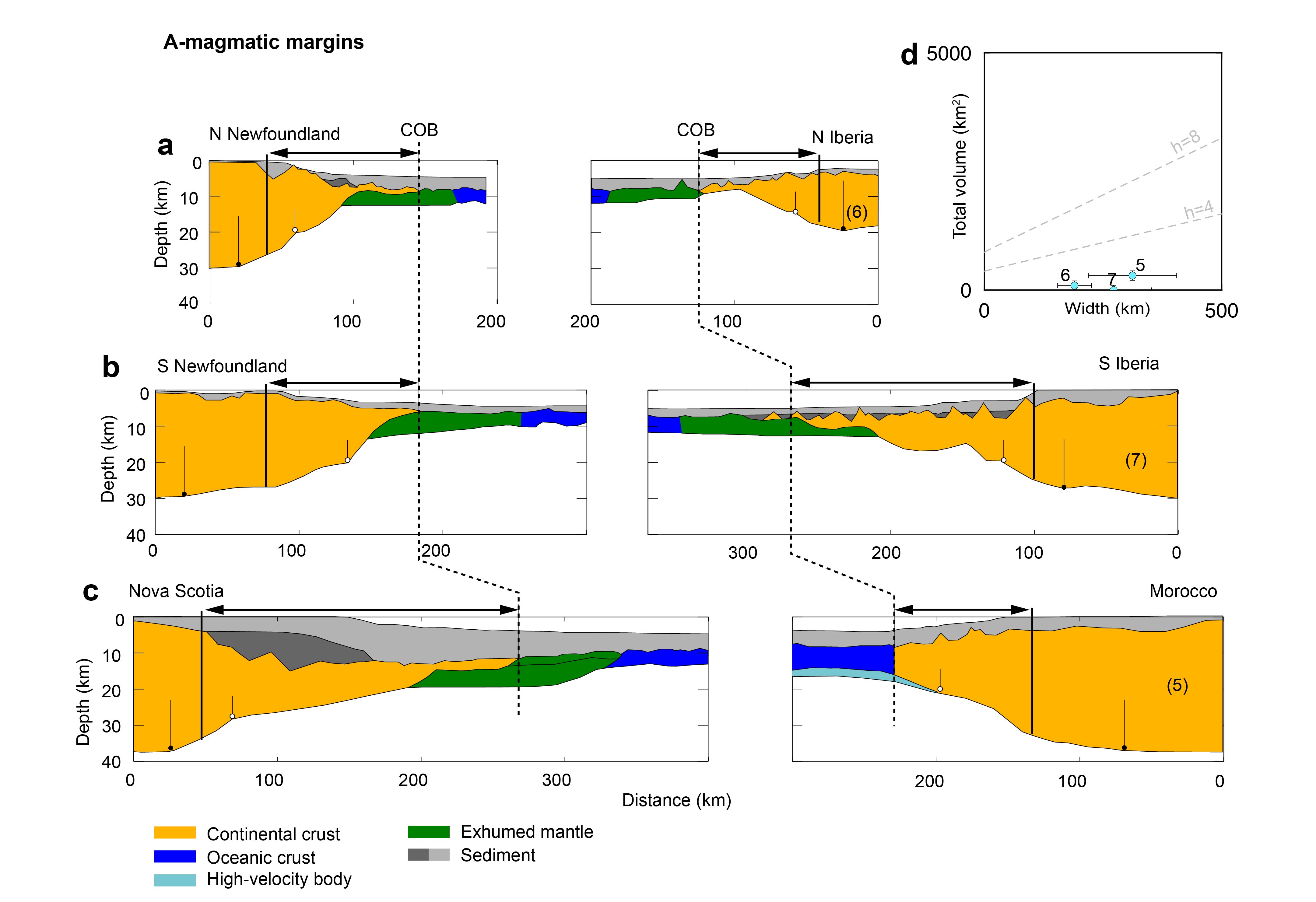
**

**Supplementary Figure 7. Melt volume measurement of a-magmatic margins.** **a-c**, Conjugate crustal cross sections for a-magmatic margins ordered increasing in width from top to bottom. Oceanic crust (blue) and exhumed a-magmatic mantle (green) are differentiated following ref. ^44^. (**a**) N. Newfoundland – N. Iberia^45–48^, (**b**) S. Newfoundland – S. Iberia^44,45,49–51^, (**c**) Morocco – Nova Scotia^24,25,50,52,53^ conjugate margins. The high-velocity body at Morocco margin is interpreted as serpentinized mantle^25,53^ or alternatively as post-rift volcanic underplate^54^. **d**, Estimated melt volume and width for each conjugate margin pair. Dashed line shows semi-analytical prediction with *h*_oc_ = 4 km and *h*_oc_ = 8 km. Note that all conjugate margin pairs shown here significantly are below the range of normal magmatic productivity for oceanic spreading systems.

Supplementary Table 1. Parameters used in thermo-mechanical models.

| Parameter | Symbol | Unit | | Value | |
| --- | --- | --- | --- | --- | --- |
| Thermal parameters | | | | | |
| Surface temperature | $T_{0}$ | °C | 0 | | |
| Moho temperature | $T_{m}$ | °C | 550 | | |
| Adiabatic thermal gradient | $\frac{dT}{dz}$ | K m^-1^ | 0.4×10^-3^ | | |
| Potential temperature | *T*_p_ | °C | 1300 | | |
| Base lithosphere temperature | *T*_l_ | °C | 1350 | | |
| Basal temperature | $T_{\mathrm{bot}}$ | °C | 1540 | | |
| Radioactive heat production | $A_{r}$ | W m^-3^ | 0.88×10^-6^ | | |
| Heat capacity | *c*_p_ | J kg^-1^ K^-1^ | 750 | | |
| Thermal conductivity | *k* | W m^-1^ K^-1^ | 2.25 | | |
| Thermal expansivity | α | K^-1^ | 2×10^-5^ | | |
| Rheological parameters | | | | | |
| Creeping laws | | | Crust  Wet Quartz^55^ | | Mantle  Wet Olivine^56^ |
| Power-law exponent | *n* | - | 4.0 | | 3.0 |
| Activation energy | *Q* | kJ mol^-1^ | 223 | | 430 |
| Activation volume | *V* | cm^3^ mol^-1^ | 0 | | 15 |
| Pre-exponential constant | *A* | Pa^-n^ s^-1^ | 1.10×10^-28^ | | 1.76×10^-14^ |
| Viscosity scaling factors | | | | | |
| Crust | *f*_c_ | - | 0.02 - 30 | | |
| Mantle lithosphere |  | - | 5 | | |
| Sub-lithospheric mantle |  | - | 1 | | |
| Plasticity | | | | | |
| Strain range of softening | $\varepsilon$ | - | 0.5 – 1.5 | | |
| Effective frictional angle | $\varphi$_eff_($\varepsilon$) | ° | 15 – 2 | | |
| Cohesion | *C* | MPa | 20 | | |
| Melting parameters | | | | | |
| Dry solidus at surface | $T_{s0}$ | °C | 1080 | | |
| Wet solidus at surface | $T_{s0w}$ | °C | 880 | | |
| Depth derivative of solidus | $\frac{{dT}_{s}}{dz}$ | K m^-1^ | 3.4×10^-3^ | | |
| Depletion derivative of solidus | $\frac{dT_{s}}{dX}$ | K | 440 | | |
| Maximum melt fraction for damp melting | $\phi_{\lim}$ | - | 0.02 | | |
| Threshold for melt retention | $\phi_{\mathrm{ret}}$ | - | 0.01 | | |
| Melt density | $\rho_{m}$ | kg m^-3^ | 2800 | | |
| Change of entropy on melting | $\Delta S$ | J kg^-1^ K^-1^ | 400 | | |
| Reference depletion | $X_{\mathrm{ref}}$ | - | 1.3 | | |
| Residual density at *X*=$X_{ref}$ | $\rho_{X_{\mathrm{ref}}}$ | kg m^-3^ | 3285 | | |
| Melt weakening constant | *a* | - | 45 | | |
| Other parameters | | | | | |
| Model thickness |  | km | 600 | | |
| Depth of base crust | *z*_m_ | km | 35 | | |
| Depth of base mantle lithosphere | *z*_l_ | km | 125 | | |
| Crustal density (at *T*=0 °C) |  | kg m^-3^ | 2800 | | |
| Mantle density (at *T*=0 °C) |  | kg m^-3^ | 3300 | | |
| Full extension velocity | *V*_ext_ | cm yr^-1^ | 1.5 | | |

**Supplementary Table 2.** Compilation of magmatic addition for North, Central, and South Atlantic rifted margins.

| id | Location | Ref. | W (km) | $W_{\max}$ (km) | $W_{\min}$ (km) | $V_{\mathrm{LCB}}$  (km${}^{2}$) | $V_{\mathrm{SDR}}^{'}$  (km${}^{2}$) | $V_{\mathrm{intrude}}^{'}$ (km${}^{2}$) | $V_{\mathrm{spread}}$ (km${}^{2}$) | $h_{50}$ (km) | $h_{25}$ (km) | $h_{10}$ (km) |
| --- | --- | --- | --- | --- | --- | --- | --- | --- | --- | --- | --- | --- |
| 1 | Brazil | ^32^ | 155 | 176.4 | 133.6 | 653$\pm$65.3 | 655.8$\pm$655.8 | 175.7$\pm$175.7 | 938.2 | 18.4 | 18.4 | 19.9 |
| 1 | Namibia | ^30^ | 161 | 190.2 | 131.8 | 611.9$\pm$61.19 | 269.2$\pm$269.2 | 150.7$\pm$150.7 | 727.8 | 12.2 | 14.6 | 16.4 |
| 2 | Argentina | ^41^ | 118.9 | 212.9 | 24.9 | 82.2$\pm$8.22 | 15.7$\pm$15.7 | 11.7$\pm$11.7 | 352.2 | 6.4 | 7 | 8.6 |
| 2 | South Africa | ^41^ | 187.3 | 208.4 | 166.2 | 609.6$\pm$60.96 | 204.4$\pm$204.4 | 62.2$\pm$62.2 | 489.7 | 8.5 | 9.8 | 10.9 |
| 3 | Argentina | ^26^ | 101 | 157.6 | 44.4 | 187.6$\pm$18.76 | 72.6$\pm$72.6 | 9.3$\pm$9.3 | 453.5 | 7.8 | 9.6 | 9.3 |
| 3 | South Africa | ^26^ | 151.8 | 174.4 | 129.2 | 526.1$\pm$52.61 | 124$\pm$124 | 87.9$\pm$87.9 | 381.3 | 6.8 | 7.4 | 8.1 |
| 4 | US East Coast | ^57^ | 166.4 | 200.3 | 132.5 | 1166.7 | 115.2$\pm$115.2 | 60.8$\pm$60.8 | 398.2 | 8.4 | 8.3 | 7.4 |
| 4 | SW Morocco | ^57^ | 54.8 | 96 | 13.6 | 113.8 | 0 | 52.8$\pm$52.8 | 533.1 | 8.6 | 9.2 | 13.7 |
| 5 | Nova Scotia | ^52^ | 218.7 | 242.3 | 195.1 | 0 | 0 | 0 | 0 | 0 | 0 | 0 |
| 5 | Morocco | ^25^ | 93.2 | 162.6 | 23.8 | 0 | 0 | 0 | 305 | 5.9 | 6.7 | 7.3 |
| 6 | Newfoundland N | ^45^ | 105.5 | 125.4 | 85.7 | 0 | 0 | 0 | 93.6 | 3.9 | 1.9 | 0 |
| 6 | Iberia N | ^45^ | 84.3 | 99.9 | 68.6 | 0 | 0 | 0 | 0 | 0 | 0 | 0 |
| 7 | Newfoundland S | ^45^ | 106.1 | 164.2 | 48.1 | 0 | 0 | 0 | 0 | 0 | 0 | 0 |
| 7 | Iberia S | ^45^ | 166.3 | 187.8 | 144.9 | 0 | 0 | 0 | 0 | 0 | 0 | 0 |
| 8 | SE Greenland | ^5^ | 61.6 | 93.9 | 29.4 | 106.8 | 90.9$\pm$90.9 | 54.4$\pm$54.4 | 691 | 11.6 | 14.11 | 15.3 |
| 8 | Edoras | ^4^ | 42 | 51.9 | 32.1 | 201.2 | 50.7$\pm$50.7 | 24.7$\pm$24.7 | 602 | 9.8 | 12.5 | 13 |
| 9 | SE Greenland | ^8^ | 60.3 | 87.2 | 33.3 | 172.5 | 54.3$\pm$54.3 | 60.7$\pm$60.7 | 859.8 | 16.9 | 17.4 | 17.7 |
| 9 | Hatton | ^9^ | 35.1 | 49.2 | 21.1 | 160.3 | 14.4$\pm$14.4 | 38.3$\pm$38.3 | 695.3 | 11.4 | 13.7 | 15.9 |
| 10 | Jan Mayen | ^12^ | 31.2 | 37.5 | 24.9 | 49.9 | 21.2$\pm$21.2 | 8.6$\pm$8.6 | 284.4 | 4.8 | 4.9 | 7.6 |
| 10 | Møre | ^2^ | 130.6 | 221.8 | 64.3 | 766.6 | 37.3$\pm$37.3 | 54.6$\pm$54.6 | 486.6 | 8.6 | 10 | 10.8 |
| 11 | NE Greenland | ^5^ | 124.4 | 147 | 101.7 | 1277.7 | 138$\pm$138 | 83.4$\pm$83.4 | 1078.8 | 15.9 | 22.4 | 23.8 |
| 11 | Vøring S | ^2^ | 166.4 | 223.4 | 103.5 | 1242.1 | 46.4$\pm$46.4 | 86.2$\pm$86.2 | 848.5 | 15.4 | 17.4 | 18 |
| 12 | NE Greenland | ^5^ | 116.9 | 140.9 | 93 | 1465.2 | 91.4$\pm$91.4 | 73.4$\pm$73.4 | 947.9 | 14.7 | 19 | 22.3 |
| 12 | Vøring N | ^2^ | 150.4 | 216.9 | 24.9 | 767.9 | 20.9$\pm$20.9 | 28.7$\pm$28.7 | 772.9 | 13.2 | 15.6 | 17.9 |
| 13 | NE Greenland | ^58^ | 96.5 | 136.5 | 56.4 | 258.1 | 87.1$\pm$87.1 | 68.3$\pm$68.3 | 392.9 | 5.6 | 7.6 | 9.9 |
| 13 | Lofoten S | ^36^ | 55.8 | 63.3 | 48.3 | 32.7 | 59.3$\pm$59.3 | 12.6$\pm$12.6 | 468.8 | 9.2 | 9.4 | 9.8 |
| 14 | NE Greenland | ^58^ | 34.4 | 49.5 | 19.3 | 31.5 | 68.5$\pm$68.5 | 18.4$\pm$18.4 | 324.2 | 5.8 | 6.4 | 7 |
| 14 | Lofoten N | ^2^ | 98.6 | 127.2 | 69.9 | 91 | 19.1$\pm$19.1 | 44.1$\pm$44.1 | 325.9 | 5.7 | 5.9 | 7.4 |

Geometrical volumes (in km${}^{2}$) for syn-rift magmatism including (1) seaward-dipping reflectors layer ($V_{\mathrm{SDR}}$), (2) high-velocity lower crustal body ($V_{\mathrm{LCB}}$), and (3) transitional intruded crust in between ($V_{\mathrm{intrude}}$) are measured. Igneous volumes of SDR and intruded crust listed in the table have been converted to pure igneous volumes following ref. ^59^ as $V_{\mathrm{SDR}}^{'}=0.5V_{\mathrm{SDR}}\pm0.5V_{\mathrm{SDR}}$ and $V_{\mathrm{intrude}}^{'}=0.1V_{\mathrm{intrude}}\pm0.1V_{\mathrm{intrude}}$, respectively. Magmatic volume over an additional 50-km spreading section ($V_{\mathrm{spread}}$) is also included. Total melt volume is calculated as $V^{*}=V_{\mathrm{LCB}}+V_{\mathrm{SDR}}^{'}+V_{\mathrm{intrude}}^{'}+V_{\mathrm{spread}}$. Uncertainty of total melt volume is given as $V_{\mathrm{err}}=0.5V_{\mathrm{SDR}}+0.1V_{\mathrm{intrude}}$. For margin sections (1), (2), and (3) where deep crust is not directly constrained by seismic reflection data, an additional uncertainty of 10% to the LCB volume (0.1$V_{\mathrm{LCB}}$) is included. $W$ is margin width in km, with uncertainties defined as $W_{\max}$ and $W_{\min}$. $h_{10}$, $h_{25}$, and $h_{50}$ are thicknesses of igneous crust at locations of 10 km, 25 km, and 50 km seaward of COB, respectively. Margin widths of Norwegian Møre, Vøring N., and Vøring S. margins are defined using last rifting phase^2^. See Supplementary Figs. 5-7 for section profiles with their ids, and Supplementary Table 3 for locations and relevant studies.

**Supplementary Table 3**: Locations of margin sections and the full list of references used in margin width and melt volume measurements.

| id | Location | Ref. | Section | Lon (${}^{\circ}$) | Lat (${}^{\circ}$) | Relevant studies |
| --- | --- | --- | --- | --- | --- | --- |
| 1 | Brazil | ^32^ | Pelotas N | -47.2 | -29.8 | ^26,31,33,60–64^ |
| 1 | Namibia | ^30^ | T4 | 11.8 | -23.6 | ^26,33,40,61,62,65,66^ |
| 2 | Argentina | ^41^ | Colorado N | -54 | -40 | ^26,33,37,39,61–63,67,68^ |
| 2 | South Africa | ^41^ | Orange N | 14.2 | -31.4 | ^26,33,39,61–63,67,68^ |
| 3 | Argentina | ^26^ | Colorado S | -58.9 | -40.8 | ^38,41–43,67,69^ |
| 3 | South Africa | ^26^ | Orange S | 17.7 | -32.8 | ^39,41,67,70^ |
| 4 | US East Coast | ^57^ | Baltimore  (LASE) | -73.6 | 38.9 | ^21,22,24,25,69,71–75^ |
| 4 | SW Morocco | ^57^ | Dakhla | -17.3 | 23 | ^21,24,25,74^ |
| 5 | Nova Scotia | ^52^ | SMART1 | -58 | 42.7 | ^24,25,50,74,76–78^ |
| 5 | Morocco | ^25^ | MIRROR1 | -9.3 | 32.6 | ^21,24,53,74,76,79,80^ |
| 6 | Newfoundland N | ^45^ | SCREECH1 | -44 | 46.6 | ^47,48,50,65,81–85^ |
| 6 | Iberia N | ^45^ | ISE1 | -9.6 | 42.2 | ^48,51,65,83^ |
| 7 | Newfoundland S | ^45^ | SCREECH2 | -45.8 | 45.8 | ^44,48–50,65,85^ |
| 7 | Iberia S | ^45^ | LG12-TGS | -10.2 | 40.6 | ^44,48,51,65,83,85^ |
| 8 | SE Greenland | ^5^ | SIGMA4 | -42.2 | 59.1 | ^3,8,13,58^ |
| 8 | Edoras | ^4^ | Cam77 | -24 | 56.6 | ^3,5,7,13,58,86,87^ |
| 9 | SE Greenland | ^8^ | SIGMA3 | -39 | 62.7 | ^3,5,9,11,13,58,88,89^ |
| 9 | Hatton | ^9^ | iSIMM | -19.3 | 59.4 | ^4,5,8,11,13,58,90^ |
| 10 | Jan Mayen | ^12^ | Jan Mayen  (P8-00) | -8 | 69 | ^5,13,58,87,91,92^ |
| 10 | Møre | ^2^ | Møre (L1) | 2.8 | 64 | ^5,13,14,58,69,87,91,93,94^ |
| 11 | NE Greenland | ^5^ | AWI20030500 | -21.6 | 73.3 | ^2,13,15,16,18–20,58,95^ |
| 11 | Vøring S | ^2^ | L2 | 5 | 66 | ^5,13,15,16,20,58,65,69,96,97^ |
| 12 | NE Greenland | ^5^ | AWI20030400 | -16.7 | 73.7 | ^2,13,15,16,58,95,98^ |
| 12 | Vøring N | ^2^ | L4 | 8.2 | 67.1 | ^5,13,58,65,69,97–99^ |
| 13 | NE Greenland | ^58^ | 6B | -14 | 74.6 | ^5,13,16,36,97,100^ |
| 13 | Lofoten S | ^36^ | LVMT2 | 11.6 | 68.4 | ^5,13,16,58,97^ |
| 14 | NE Greenland | ^58^ | 7B | -7.5 | 75.7 | ^13,16,36,97,100^ |
| 14 | Lofoten N | ^2^ | L6 | 13 | 69 | ^4,13,16,35,36,58,97^ |

# Lon. and Lat. are in longitude and latitude, respectively. See Supplementary Figs. 5-7 for section profiles with their ids.

# Supplementary References

1. Grevemeyer, I., Ranero, C. R. & Ivandic, M. Structure of oceanic crust and serpentinization at subduction trenches. *Geosphere* **14**, 395–418 (2018).

2. Faleide, J. I. *et al.* Structure and evolution of the continental margin off Norway and the Barents Sea. *Episodes* **31**, 82–91 (2008).

3. Holbrook, W. S. S. *et al.* Mantle thermal structure and active upwelling during continental breakup in the North Atlantic. *Earth Planet. Sci. Lett.* **190**, 251–266 (2001).

4. Barton, A. J. & White, R. S. Crustal structure of Edoras Bank continental margin and mantle thermal anomalies beneath the North Atlantic. *J. Geophys. Res. Solid Earth* **102**, 3109–3129 (1997).

5. Funck, T. *et al.* A review of the NE Atlantic conjugate margins based on seismic refraction data. *Geol. Soc. Spec. Publ.* **447**, 171–205 (2017).

6. Tsikalas, F., Faleide, J. I., Eldholm, O. & Antonio Blaich, O. The NE Atlantic conjugate margins. in *Regional Geology and Tectonics: Phanerozoic Passive Margins, Cratonic Basins and Global Tectonic Maps* 140–201 (Elsevier, 2012). doi:10.1016/B978-0-444-56357-6.00004-4

7. Barton, A. J. & White, R. S. The Edoras Bank margin; continental break-up in the presence of a mantle plume. *J. - Geol. Soc.* **152**, 971–974 (1995).

8. Hopper, J. R. *et al.* Structure of the SE Greenland margin from seismic reflection and refraction data: Implications for nascent spreading center subsidence and asymmetric crustal accretion during North Atlantic opening. *J. Geophys. Res. Solid Earth* **108**, B5 (2003).

9. White, R. S. & Smith, L. K. Crustal structure of the Hatton and the conjugate east Greenland rifted volcanic continental margins, NE Atlantic. *J. Geophys. Res. Solid Earth* **114**, B02305 (2009).

10. Fowler, S. R., White, R. S., Spence, G. D. & Westbrook, G. K. The Hatton Bank continental margin--II. Deep structure from two-ship expanding spread seismic profiles. *Geophys. J. Int.* **96**, 295–309 (1989).

11. White, R. S. *et al.* Lower-crustal intrusion on the North Atlantic continental margin. *Nature* **452**, 460–464 (2008).

12. Breivik, A. J., Mjelde, R., Faleide, J. I. & Murai, Y. The eastern Jan Mayen microcontinent volcanic margin. *Geophys. J. Int.* **188**, 798–818 (2012).

13. Gernigon, L. *et al.* Crustal fragmentation, magmatism, and the diachronous opening of the Norwegian-Greenland Sea. *Earth-Science Rev.* **206**, 102839 (2020).

14. Breivik, A. J., Mjelde, R., Faleide, J. I. & Murai, Y. Rates of continental breakup magmatism and seafloor spreading in the Norway Basin–Iceland plume interaction. *J. Geophys. Res.* **111**, 1–17 (2006).

15. Voss, M. & Jokat, W. Continent-ocean transition and voluminous magmatic underplating derived from P-wave velocity modelling of the East Greenland continental margin. *Geophys. J. Int.* **170**, 580–604 (2007).

16. Voss, M., Schmidt-Aursch, M. C. & Jokat, W. Variations in magmatic processes along the East Greenland volcanic margin. *Geophys. J. Int.* **177**, 755–782 (2009).

17. Breivik, A. J., Faleide, J. I., Mjelde, R. & Flueh, E. R. Magma productivity and early seafloor spreading rate correlation on the northern Vøring Margin, Norway—Constraints on mantle melting. *Tectonophysics* **468**, 206–223 (2009).

18. Breivik, A. J., Faleide, J. I. & Mjelde, R. Neogene magmatism northeast of the Aegir and Kolbeinsey ridges, NE Atlantic: Spreading ridge-mantle plume interaction? *Geochemistry, Geophys. Geosystems* **9**, Q02004 (2008).

19. Breivik, A., Faleide, J. I., Mjelde, R., Flueh, E. & Murai, Y. Magmatic development of the outer Vøring margin from seismic data. *J. Geophys. Res. Solid Earth* **119**, 6733–6755 (2014).

20. Mjelde, R., Kvarven, T., Faleide, J. I. & Thybo, H. Lower crustal high-velocity bodies along North Atlantic passive margins, and their link to Caledonian suture zone eclogites and Early Cenozoic magmatism. *Tectonophysics* **670**, 16–29 (2016).

21. Klingelhoefer, F. *et al.* Crustal structure of the SW-Moroccan margin from wide-angle and reflection seismic data (the DAKHLA experiment) Part A: Wide-angle seismic models. *Tectonophysics* **468**, 63–82 (2009).

22. LASE Study Group. Deep structure of the US East Coast passive margin from large aperture seismic experiments (LASE). *Mar. Pet. Geol.* **3**, 234–242 (1986).

23. Labails, C., Olivet, J.-L. L. & Group, D. S. Crustal structure of the SW Moroccan margin from wide-angle and reflection seismic data (the Dakhla experiment). Part B---The tectonic heritage. *Tectonophysics* **468**, 83–97 (2009).

24. Biari, Y. *et al.* Opening of the central Atlantic Ocean: Implications for geometric rifting and asymmetric initial seafloor spreading after continental breakup. *Tectonics* **36**, 1129–1150 (2017).

25. Klingelhoefer, F. *et al.* Crustal structure variations along the NW-African continental margin: A comparison of new and existing models from wide-angle and reflection seismic data. *Tectonophysics* **674**, 227–252 (2016).

26. Blaich, O. A., Faleide, J. I., Tsikalas, F., Gordon, A. C. & Mohriak, W. Crustal-scale architecture and segmentation of the South Atlantic volcanic margin. *Geol. Soc. London, Spec. Publ.* **369**, 167–183 (2013).

27. Contrucci, I. *et al.* Deep structure of the West African continental margin (Congo, Zaïre, Angola), between 5°S and 8°S, from reflection/refraction seismics and gravity data. *Geophys. J. Int.* **158**, 529–553 (2004).

28. Bauer, K. *et al.* Deep structure of the Namibia continental margin as derived from integrated geophysical studies. *J. Geophys. Res. Solid Earth* **105**, 25829–25853 (2000).

29. Taposeea, C. A., Armitage, J. J. & Collier, J. S. Asthenosphere and lithosphere structure controls on early onset oceanic crust production in the southern South Atlantic. *Tectonophysics* **716**, 4–20 (2017).

30. Gladczenko, T. P., Skogseid, J. & Eldhom, O. Namibia volcanic margin. *Mar. Geophys. Res.* **20**, 313–341 (1998).

31. McDermott, K., Gillbard, E. & Clarke, N. From Basalt to Skeletons--the 200 million-year history of the Namibian margin uncovered by new seismic data. *First Break* **33**, 77–85 (2015).

32. Stica, J. M., Zalán, P. V. & Ferrari, A. L. The evolution of rifting on the volcanic margin of the Pelotas Basin and the contextualization of the Paraná--Etendeka LIP in the separation of Gondwana in the South Atlantic. *Mar. Pet. Geol.* **50**, 1–21 (2014).

33. Blaich, O. A., Faleide, J. I. & Tsikalas, F. Crustal breakup and continent-ocean transition at South Atlantic conjugate margins. *J. Geophys. Res. Solid Earth* **116**, 1–38 (2011).

34. Tsikalas, F., Eldholm, O. & Faleide, J. I. Early Eocene sea floor spreading and continent-ocean boundary between Jan Mayen and Senja fracture zones in the Norwegian-Greenland Sea. *Mar. Geophys. Res.* **23**, 247–270 (2002).

35. Breivik, A. J., Faleide, J. I., Mjelde, R., Flueh, E. R. & Murai, Y. A new tectono-magmatic model for the Lofoten/Vesterålen Margin at the outer limit of the Iceland Plume influence. *Tectonophysics* **718**, 25–44 (2017).

36. Tsikalas, F., Eldholm, O. & Faleide, J. I. Crustal structure of the Lofoten-Vesterålen continental margin, off Norway. *Tectonophysics* **404**, 151–174 (2005).

37. Franke, D. *et al.* Crustal structure across the Colorado Basin, offshore Argentina. *Geophys. J. Int.* **165**, 850–864 (2006).

38. Schnabel, M. *et al.* The structure of the lower crust at the Argentine continental margin, South Atlantic at 44°S. *Tectonophysics* **454**, 14–22 (2008).

39. Hirsch, K. K., Bauer, K. & Scheck-Wenderoth, M. Deep structure of the western South African passive margin - Results of a combined approach of seismic, gravity and isostatic investigations. *Tectonophysics* **470**, 57–70 (2009).

40. Koopmann, H. *et al.* Segmentation and volcano-tectonic characteristics along the SW African continental margin, South Atlantic, as derived from multichannel seismic and potential field data. *Mar. Pet. Geol.* **50**, 22–39 (2014).

41. Blaich, O. A., Faleide, J. I., Tsikalas, F., Franke, D. & León, E. Crustal-scale architecture and segmentation of the Argentine margin and its conjugate off South Africa. *Geophys. J. Int.* **178**, 85–105 (2009).

42. Franke, D., Neben, S., Ladage, S., Schreckenberger, B. & Hinz, K. Margin segmentation and volcano-tectonic architecture along the volcanic margin off Argentina/Uruguay, South Atlantic. *Mar. Geol.* **244**, 46–67 (2007).

43. Franke, D. *et al.* Birth of a volcanic margin off Argentina, South Atlantic. *Geochemistry, Geophys. Geosystems* **11**, Q0AB04 (2010).

44. Péron-Pinvidic, G. & Manatschal, G. The final rifting evolution at deep magma-poor passive margins from Iberia-Newfoundland: A new point of view. *Int. J. Earth Sci. (Geol. Rundsch.)* **98**, 1581–1597 (2009).

45. Sutra, E., Manatschal, G., Mohn, G. & Unternehr, P. Quantification and restoration of extensional deformation along the Western Iberia and Newfoundland rifted margins. *Geochemistry, Geophys. Geosystems* **14**, 2575–2597 (2013).

46. Funck, T. *et al.* Crustal structure of the ocean-continent transition at Flemish Cap: Seismic refraction results. *J. Geophys. Res. Solid Earth* **108**, B11 (2003).

47. Hopper, J. R., Funck, T. & Tucholke, B. E. Structure of the Flemish Cap margin, Newfoundland: Insights into mantle and crustal processes during continental breakup. *Geol. Soc. Spec. Publ.* **282**, 47–61 (2007).

48. Peron-Pinvidic, G., Manatschal, G. & Osmundsen, P. T. Structural comparison of archetypal Atlantic rifted margins: A review of observations and concepts. *Mar. Pet. Geol.* **43**, 21–47 (2013).

49. Van Avendonk, H. J. A. A. *et al.* Seismic velocity structure of the rifted margin of the eastern Grand Banks of Newfoundland, Canada. *J. Geophys. Res. Solid Earth* **111**, B11404 (2006).

50. Minshull, T. A. Geophysical characterisation of the ocean-continent transition at magma-poor rifted margins. *Comptes Rendus Geosci.* **341**, 382–393 (2009).

51. Sutra, E. & Manatschal, G. How does the continental crust thin in a hyperextended rifted margin? Insights from the Iberia margin. *Geology* **40**, 139–142 (2012).

52. Funck, T., Jackson, H. R., Louden, K. E., Dehler, S. A. & Wu, Y. Crustal structure of the northern Nova Scotia rifted continental margin (eastern Canada). *J. Geophys. Res. Solid Earth* **109**, B09102 (2004).

53. Biari, Y. *et al.* Deep crustal structure of the North-West African margin from combined wide-angle and reflection seismic data (MIRROR seismic survey). *Tectonophysics* **656**, 154–174 (2015).

54. Holik, J. S., Rabinowitz, P. D. & Austin, J. A. Effects of Canary hotspot volcanism on structure of oceanic crust off Morocco. *J. Geophys. Res.* **96**, 12039–12067 (1991).

55. Gleason, G. C. & Tullis, J. A flow law for dislocation creep of quartz aggregates determined with the molten salt cell. *Tectonophysics* **247**, 1–23 (1995).

56. Karato, S. I. & Wu, P. Rheology of the upper mantle: A synthesis. *Science (80-. ).* **260**, 771–778 (1993).

57. Labails, C., Olivet, J.-L. & Group, D. S. Crustal structure of the SW Moroccan margin from wide-angle and reflection seismic data (the Dakhla experiment). Part B---The tectonic heritage. *Tectonophysics* **468**, 83–97 (2009).

58. Tsikalas, F., Faleide, J. I., Eldholm, O., Antonio Blaich, O. & Blaich, O. A. The NE Atlantic conjugate margins. *Reg. Geol. Tectonics Phaneroz. Passiv. Margins, Cratonic Basins Glob. Tecton. Maps* **1**, 140–201 (2012).

59. Voss, M. & Jokat, W. From Devonian extensional collapse to early Eocene continental break-up: An extended transect of the Kejser Franz Joseph Fjord of the East Greenland margin. *Geophys. J. Int.* **177**, 743–754 (2009).

60. Geoffroy, L., Burov, E. B. & Werner, P. Volcanic passive margins: Another way to break up continents. *Sci. Rep.* **5**, 1–12 (2015).

61. Heine, C., Zoethout, J. & Müller, R. D. Kinematics of the South Atlantic rift. *Solid Earth* **4**, 215–253 (2013).

62. Maurya, V. P., Fontes, S. L., Oliveira, V. C. & La Terra, E. F. Gradient based first- and second-order filters for the demarcation of continental-oceanic boundaries using satellite gravity data. *Geophys. J. Int.* **221**, 1499–1514 (2020).

63. Reuber, K., Mann, P. & Pindell, J. Hotspot origin for asymmetrical conjugate volcanic margins of the austral South Atlantic Ocean as imaged on deeply penetrating seismic reflection lines. *Interpretation* **7**, SH71–SH97 (2019).

64. Müller, R. D. *et al.* Ocean Basin Evolution and Global-Scale Plate Reorganization Events since Pangea Breakup. *Annu. Rev. Earth Planet. Sci.* **44**, 107–138 (2016).

65. Clerc, C., Ringenbach, J. C., Jolivet, L. & Ballard, J. F. Rifted margins: Ductile deformation, boudinage, continentward-dipping normal faults and the role of the weak lower crust. *Gondwana Res.* **53**, 20–40 (2018).

66. Strozyk, F., Back, S. & Kukla, P. A. Comparison of the rift and post-rift architecture of conjugated salt and salt-free basins offshore Brazil and Angola/Namibia, South Atlantic. *Tectonophysics* **716**, 204–224 (2017).

67. Becker, K. *et al.* Asymmetry of high-velocity lower crust on the South Atlantic rifted margins and implications for the interplay of magmatism and tectonics in continental breakup. *Solid Earth* **5**, 1011–1026 (2014).

68. Paton, D. A., Pindell, J., McDermott, K., Bellingham, P. & Horn, B. Evolution of seaward-dipping reflectors at the onset of oceanic crust formation at volcanic passive margins: Insights from the South Atlantic. *Geology* **45**, 439–442 (2017).

69. Eldholm, O., Gladczenko, T. P., Skogseid, J. & Planke, S. Atlantic volcanic margins: A comparative study. *Geol. Soc. Lond. Spec. Publ.* **167**, 411–428 (2000).

70. Koopmann, H., Schreckenberger, B., Franke, D., Becker, K. & Schnabel, M. The late rifting phase and continental break-up of the southern South Atlantic: the mode and timing of volcanic rifting and formation of earliest oceanic crust. *Geol. Soc. Lond. Spec. Publ.* **420**, 315–340 (2014).

71. Talwani, M. & Abreu, V. Inferences regarding initiation of oceanic crust formation from the US East Coast margin and conjugate South Atlantic margins. *Geophys. Monogr. Geophys. Union* **115**, 211–234 (2000).

72. Holbrook, W. S. & Kelemen, P. B. Large igneous province on the US Atlantic margin and implications for magmatism during continental breakup. *Nature* **364**, 433–436 (1993).

73. Holbrook, W. S. Seismic structure of the US Mid-Atlantic continental margin. *J. Geophys. Res.* **99**, 17871–17891 (1994).

74. Labails, C., Olivet, J. L., Aslanian, D. & Roest, W. R. An alternative early opening scenario for the Central Atlantic Ocean. *Earth Planet. Sci. Lett.* **297**, 355–368 (2010).

75. Kelemen, P. B. & Holbrook, W. S. Origin of thick, high-velocity igneous crust along the US east coast margin. *J. Geophys. Res.* **100**, (1995).

76. Louden, K., Wu, Y. & Tari, G. Systematic variations in basement morphology and rifting geometry along the nova scotia and morocco conjugate margins. *Geol. Soc. Spec. Publ.* **369**, 267–287 (2013).

77. Wu, Y., Louden, K. E., Funck, T., Jackson, H. R. & Dehler, S. A. Crustal structure of the central Nova Scotia margin off Eastern Canada. *Geophys. J. Int.* **166**, 878–906 (2006).

78. Keen, C. E. & Potter, D. P. The transition from a volcanic to a nonvolcanic rifted margin off eastern Canada. *Tectonics* **14**, 359–371 (1995).

79. Contrucci, I. *et al.* The crustal structure of the NW Moroccan continental margin from wide-angle and reflection seismic data. *Geophys. J. Int.* **159**, 117–128 (2004).

80. Jaffal, M., Klingelhoefer, F., Matias, L., Teixeira, F. & Amrhar, M. Crustal structure of the NW Moroccan margin from deep seismic data (SISMAR Cruise). *Comptes Rendus - Geosci.* **341**, 495–503 (2009).

81. Funck, T., Jackson, H. R., Louden, K. E. & Klingelhöfer, F. Seismic study of the transform-rifted margin in Davis Strait between Baffin Island (Canada) and Greenland: What happens when a plume meets a transform. *J. Geophys. Res. Solid Earth* **112**, 1–22 (2007).

82. Hopper, J. R. *et al.* Continental breakup and the onset of ultraslow seafloor spreading off Flemish Cap on the Newfoundland rifted margin. *Geology* **32**, 93–96 (2004).

83. Dean, S. L., Sawyer, D. S. & Morgan, J. K. Galicia Bank ocean-continent transition zone: new seismic reflection constraints. *Earth Planet. Sci. Lett.* **413**, 197–207 (2015).

84. Hopper, J. R. *et al.* A deep seismic investigation of the Flemish Cap margin: Implications for the origin of deep reflectivity and evidence for asymmetric break-up between Newfoundland and Iberia. *Geophys. J. Int.* **164**, 501–515 (2006).

85. Lau, K. W. H. *et al.* Crustal structure across the Grand Banks---Newfoundland Basin Continental Margin---I. Results from a seismic refraction profile. *Geophys. J. Int.* **167**, 127–156 (2006).

86. Collier, J. S. *et al.* Factors influencing magmatism during continental breakup: New insights from a wide-angle seismic experiment across the conjugate Seychelles-Indian margins. *J. Geophys. Res.* **114**, B03101 (2009).

87. Mjelde, R., Raum, T., Breivik, A. J. & Faleide, J. I. Crustal transect across the North Atlantic. *Mar. Geophys. Res.* **29**, 73–87 (2008).

88. Smith, L. K., White, R. S. & Kusznir, N. J. Structure of the Hatton Basin and adjacent continental margin. *Geol. Soc. Lond. Pet. Geol. Conf. Ser.* **6**, 947–956 (2005).

89. Horni, J. *et al.* Regional distribution of volcanism within the North Atlantic Igneous Province. *Geol. Soc. Spec. Publ.* **447**, 105–125 (2017).

90. White, R. S. Magmatism during and after continental break-up. *Geol. Soc. Lond. Spec. Publ.* **68**, 1–16 (1992).

91. Kvarven, T. *et al.* Crustal composition of the Møre Margin and compilation of a conjugate Atlantic margin transect. *Tectonophysics* **666**, 144–157 (2016).

92. Kodaira, S., Mjelde, R., Gunnarsson, K., Shiobara, H. & Shimamura, H. Structure of the Jan Mayen microcontinent and implications for its evolution. *Geophys. J. Int.* **132**, 383–400 (1998).

93. Eccles, J. D., White, R. S. & Christie, P. A. F. The composition and structure of volcanic rifted continental margins in the North Atlantic: Further insight from shear waves. *Tectonophysics* **508**, 22–33 (2011).

94. Theissen-Krah, S. *et al.* Tectonic evolution and extension at the Møre Margin--Offshore mid-Norway. *Tectonophysics* **721**, 227–238 (2017).

95. Geissler, W. H. *et al.* Seismic volcanostratigraphy of the NE Greenland continental margin. *Geol. Soc. Spec. Publ.* **447**, 149–170 (2017).

96. Neumann, E. *et al.* Sill and lava geochemistry of the mid-Norway and NE Greenland conjugate margins. *Geochem., Geophys. Geosys.* **14**, 3666–3690 (2013).

97. Tsikalas, F., Faleide, J. I. & Kusznir, N. J. Along-strike variations in rifted margin crustal architecture and lithosphere thinning between northern Vøring and Lofoten margin segments off mid-Norway. *Tectonophysics* **458**, 68–81 (2008).

98. Breivik, A. J., Faleide, J. I., Mjelde, R. & Flueh, E. R. Magma productivity and early seafloor spreading rate correlation on the northern Vøring Margin, Norway—Constraints on mantle melting. *Tectonophysics* **468**, 206–223 (2009).

99. Mjelde, R., Raum, T., Kandilarov, A., Murai, Y. & Takanami, T. Crustal structure and evolution of the outer Møre Margin, NE Atlantic. *Tectonophysics* **468**, 224–243 (2009).

100. Olesen, O. *et al.* An improved tectonic model for the Eocene opening of the Norwegian-Greenland Sea: Use of modern magnetic data. *Mar. Pet. Geol.* **24**, 53–66 (2007).
